# Characterization and Clinical Impact of Bloodstream Infection Caused by Carbapenemase-Producing *Enterobacteriaceae* in Seven Latin American Countries

**DOI:** 10.1371/journal.pone.0154092

**Published:** 2016-04-22

**Authors:** Maria Virginia Villegas, Christian J. Pallares, Kevin Escandón-Vargas, Cristhian Hernández-Gómez, Adriana Correa, Carlos Álvarez, Fernando Rosso, Lorena Matta, Carlos Luna, Jeannete Zurita, Carlos Mejía-Villatoro, Eduardo Rodríguez-Noriega, Carlos Seas, Manuel Cortesía, Alfonso Guzmán-Suárez, Manuel Guzmán-Blanco

**Affiliations:** 1 International Center for Medical Research and Training (CIDEIM), Cali, Colombia; 2 Hospital Universitario del Valle Evaristo García E.S.E., Cali, Colombia; 3 Hospital Universitario San Ignacio, Facultad de Medicina, Universidad Nacional de Colombia, Bogotá, Colombia; 4 Clínica Fundación Valle del Lili, Cali, Colombia; 5 Pontificia Universidad Javeriana, Cali, Colombia; 6 Hospital de Clínicas José de San Martín, Buenos Aires, Argentina; 7 Hospital Vozandes, Facultad de Medicina, Pontificia Universidad Católica del Ecuador, Quito, Ecuador; 8 Hospital Roosevelt, Guatemala City, Guatemala; 9 Hospital Civil de Guadalajara Fray Antonio Alcalde, Guadalajara, Mexico; 10 Hospital Nacional Cayetano Heredia, Lima, Peru; 11 Hospital de Clínicas Caracas, Caracas, Venezuela; 12 Centro Médico de Caracas, Caracas, Venezuela; Curtin University, AUSTRALIA

## Abstract

**Introduction:**

Infections caused by carbapenem-resistant *Enterobacteriaceae* are a public health problem associated with higher mortality rates, longer hospitalization and increased healthcare costs. We carried out a study to describe the characteristics of patients with carbapenemase-producing *Enterobacteriaceae* (CPE) and non-CPE bloodstream infection (BSI) from Latin American hospitals and to determine the clinical impact in terms of mortality and antibiotic therapy.

**Methods:**

Between July 2013 and November 2014, we conducted a multicenter observational study in 11 hospitals from 7 Latin American countries (Argentina, Colombia, Ecuador, Guatemala, Mexico, Peru, Venezuela). Patients with BSI caused by *Enterobacteriaceae* were included and classified either as CPE or non-CPE based on detection of *bla*_KPC_, *bla*_VIM_, *bla*_IMP_, *bla*_NDM_ and *bla*_OXA-48_ by polymerase chain reaction.

Enrolled subjects were followed until discharge or death. Demographic, microbiological and clinical characteristics were collected from medical records. Both descriptive and inferential statistics were used to analyze the information.

**Results:**

A total of 255 patients with *Enterobacteriaceae* BSI were included; CPE were identified in 53 of them. *In vitro* non-susceptibility to all screened antibiotics was higher in the patients with CPE BSI, remaining colistin, tigecycline and amikacin as the most active drugs. Combination therapy was significantly more frequent in the CPE BSI group (*p* < 0.001). The most common regimen was carbapenem + colistin or polymyxin B. The overall mortality was 37% (94/255). Overall and attributable mortality were significantly higher in patients with CPE BSI (*p* < 0.001); however, we found that patients with CPE BSI who received combination therapy and those who received monotherapy had similar mortality. After multivariate adjustment, CPE BSI (adjusted odds ratio [aOR] 4; 95% confidence interval [CI] 1.7–9.5; *p* = 0.002) and critical illness (aOR 6.5; 95% CI 3.1–13.7; *p* < 0.001) were independently associated with in-hospital mortality.

**Conclusions:**

This study provides valuable data on the clinical characteristics and mortality risk factors in patients with CPE BSI. We determined that CPE infection is an independent mortality predictor and thus Latin American hospitals should perform campaigns on prevention and control of CPE BSI.

## Introduction

Infections caused by carbapenem-resistant *Enterobacteriaceae* (CRE) have been increasingly reported in the last decade and have become a public health problem of global dimensions [1–3]. Carbapenemases constitute the major mechanism of carbapenems and most other β-lactam antibiotics resistance in *Enterobacteriaceae* [1,4,5]. CRE are also commonly associated with additional mechanisms of resistance to other antibiotic classes, including aminoglycosides and fluoroquinolones [6,7]. As a result of this broad-spectrum antibiotic resistance, very limited options for treating CRE infections are available [1].

Furthermore, CRE infections are associated with higher mortality rates, longer hospitalizations and increased healthcare costs [1,8]. CRE are a cause of many types of infections, including bloodstream, central venous catheter-related, urinary tract, surgical site, respiratory tract, and intra-abdominal infections [9]. Bloodstream infection (BSI) is the major clinical syndrome caused by CRE, accounting for the majority of CRE infections [10,11]. Several studies in the USA, Greece, Italy, Israel, Spain and Brazil have reported crude fatality rates from 24% to as high as 72% in patients with CRE BSI [11–21]. In a pooled analysis including studies up to 2012, Falagas *et al* reported mortality twice as high in patients with CRE BSI compared to carbapenem-susceptible *Enterobacteriaceae* BSI (relative risk [RR] 2.2; 95% confidence interval [CI] 1.8–2.6) [8].

While previous Latin American studies have focused on the molecular characterization of carbapenemases in *Enterobacteriaceae* and its epidemiological surveillance [5,22,23], few regional studies on the clinical impact of carbapenemase-producing *Enterobacteriaceae* (CPE) BSI have been performed [18]. In this study, we aimed to describe the clinical characteristics of patients with CPE and non-CPE BSI from Latin American hospitals, and to determine the clinical impact in terms of in-hospital mortality and antibiotic therapy.

## Methods

### Setting

The study was conducted between July 2013 and November 2014 in 11 high-complexity medical centers from Argentina, Colombia, Ecuador, Guatemala, Mexico, Peru, and Venezuela.

### Study design and population

This was a multicenter observational study that included consecutive inpatients of any age and sex with BSI caused by any *Enterobacteriaceae* with minimal inhibitory concentration (MIC) ≥ 1 μg/mL for cefotaxime and/or ceftriaxone. Only one isolate per patient was included. Individuals included had clinical findings of systemic inflammatory response syndrome [24]. Exclusion criteria were patients whose isolates grew more than one microorganism or whose patient’s clinical data were incomplete. Patients with CPE BSI were designated as exposed patients, whereas patients with non-CPE BSI were designated as non-exposed. CPE was defined based on the molecular detection by polymerase chain reaction (PCR) of any carbapenemase (described ahead).

### Sample size and sampling

Sample size was calculated using Stata^®^ version 9.0 (StataCorp LP, College Station, TX, USA). We considered a mortality rate of 32.1% in patients with CPE infection and of 9.9% in patients with non-CPE infection [25], a *bla*_KPC_ prevalence of 12.8% in gram-negative bacteria [26], a 95% confidence level and an 80% power. Ratio of exposed to non-exposed patients was ≈1:4.

### Data collection

Enrolled subjects who met selection criteria were followed until discharge or death. Demographic, microbiological and clinical characteristics (i.e., age, sex, acquisition of infection, bacterial isolate, bacteremia source, Pitt bacteremia score (PBS) [27], intensive care unit [ICU] admission, underlying diseases and comorbidities, and treatment of the bacteremic episode) were collected from medical records into a case report form (CRF). Blood samples obtained during hospitalization were used for microbiological cultures. All microbiology laboratories of the participating hospitals used automated systems for microorganism identification and *in vitro* antibiotic susceptibility testing. Antimicrobial MICs were interpreted according to the breakpoints of the Clinical and Laboratory Standards Institute (CLSI) 2014 guidelines [28]. In each hospital one co-investigator and one coordinator were in charge of the patient selection and follow-up, as well as of the data collection, and shipment of isolates and CRFs to the International Center for Medical Research and Training (CIDEIM).

### Definitions

Bacteremia onset was defined as the date of blood culture collection. BSI was classified as hospital- or community-acquired according to the time since hospital admission (i.e., ≥ 48 hours and < 48 hours, respectively). Bacteremia source was identified using the Centers for Disease Control and Prevention (CDC) criteria [29]. The PBS, a standardized severity-of-illness scoring system based on body temperature, blood pressure, mental status, mechanical ventilation and cardiac arrest [27], was calculated at the moment of blood culture collection. Critical illness was defined as a PBS ≥ 4. Antibiotics were selected by the attending physician in each case. Empirical treatment was defined as the antibiotics given before knowing the bacterial identification and susceptibility report provided by the hospital laboratory. Antibiotic treatment given in accordance with the culture result was deemed as definitive, if received for at least 48 hours. Treatment regimens were classified either as monotherapy (one *in vitro* active antibiotic) or combination therapy (two or more *in vitro* active antibiotics). We determined the final outcome as discharge or all-cause death at 72 hours, 7 days and 28 days since bacteremia onset. Patients discharged before the respective cutoff were considered survivors. Mortality attributable to BSI was defined as the death of a patient with clinical and laboratory evidence of ongoing infection in absence of other feasible reasons.

### Microbiological procedures

In CIDEIM, bacterial identification for isolates was confirmed using the Vitek-2 automated system (bioMérieux, Marcy-l’Étoile, France). Molecular characterization for *bla*_KPC_, *bla*_VIM_, *bla*_IMP_, *bla*_NDM_, and *bla*_OXA-48_ was performed by real-time PCR as previously described [30,31].

### Statistical analysis

We conducted database processing and analysis in Stata^®^ version 9.0 (idem) (Dataset in S1 Dataset). Prior to analysis, data was anonymized, de-identified and aggregated. We used relative frequencies for categorical variables, and central tendency and dispersion measures for numerical variables. Data were analyzed between exposed and non-exposed subjects using Chi-square (*χ*^*2*^) test or Fisher exact test for categorical variables, and Student *t* test or Mann-Whitney *U* test for numerical variables, as appropriate. All *p* values were deemed as statistically significant when < 0.05. Time-to-event analysis was performed through Kaplan-Meier estimates between groups, and these were compared by the log rank test at 72 hours, 7 days and 28 days after bacteremia onset. Moreover, variables included in the multivariate logistic regression model were those with *p* value < 0.2 in bivariate analysis (critical illness, change of the empirical regimen after culture report, definitive treatment with carbapenem, CPE BSI). Independent predictors of in-hospital mortality were sought. Odds ratios (ORs) and their 95% CIs were calculated.

### Ethical approval

The study was performed in accordance with the 1964 Declaration of Helsinki and its later amendments. The Institutional Review Board of the International Center for Medical Research (CIDEIM), Cali, Colombia, and the Ethics Committees of the hospitals (Hospital Universitario del Valle Evaristo García E.S.E., Clínica Fundación Valle del Lili, Cali, Colombia, Hospital Universitario San Ignacio, Hospital de Clínicas José de San Martín, Hospital Vozandes, Hospital de las Fuerzas Armadas de Quito, Hospital Roosevelt, Hospital Civil de Guadalajara Fray Antonio Alcalde, Hospital Nacional Cayetano Heredia, Hospital de Clínicas Caracas, and Centro Médico de Caracas) approved the study. The Institutional Review Board of CIDEIM waived the need for written or verbal informed consent from each study participant considering the minimal risk of this research.

## Results

### General characteristics of patients and isolates

Six hundred and eighty-six *Enterobacteriaceae* blood isolates were collected and sent to CIDEIM, among which 255/686 (37%) with their corresponding patients’ CRFs met selection criteria and hence were included in the study. Distribution per country of *Enterobacteriaceae* isolates and CRFs included is shown in Table 1. CPE were identified in 53/255 (21%) of all individuals; the other 202 patients had non-CPE isolates (Table 2). Out of the 53 CPE isolates, 44 (83%) harbored *bla*_KPC_, 5 (9%) harbored *bla*_VIM_, and 4 (8%) harbored *bla*_NDM_. None of the isolates were positive for IMP or OXA-48. Interestingly, all NDM isolates were from Guatemala and all VIM isolates were from Mexico.

**Table 1:** Distribution per country of *Enterobacteriaceae* isolates and case report forms from patients included in the study.

Country	Cities	Hospitals, No.	Patients, No. (%)	Patients with CPE BSI, No.
Argentina	Buenos Aires	1	24 (9%)	9^a^
Colombia	Bogotá and Cali	3	128 (50%)	32^a^
Ecuador	Quito	2	8 (3%)	1^a^
Guatemala	Guatemala City	1	20 (8%)	4^b^
Mexico	Guadalajara	1	25 (10%)	5^c^
Peru	Lima	1	40 (16%)	0
Venezuela	Caracas	2	10 (4%)	2^a^
**Total**	8	11	255 (100%)	53/255

CPE BSI, carbapenemase-producing *Enterobacteriaceae* bloodstream infection.

^a^, KPC-producing bacteria.

^b^, NDM-producing bacteria.

^c^, VIM-producing bacteria.

**Table 2:** Comparison of selected variables between patients with carbapenemase-producing *Enterobacteriaceae* (CPE) bloodstream infection (BSI) and patients with non-CPE BSI.

	Patients, No. (%)^a^		
Variable	Patients with CPE BSI, *n* = 53	Patients with non-CPE BSI, *n* = 202	*p* value
Age, median (range), y	59 (0.1–91)	60 (0.1–99)	0.80^b^
Sex (*n* = 248)			0.41^c^
Male	33 (63%)	112 (57%)	
Female	19 (37%)	84 (43%)	
Acquisition of infection			0.01^c^
Hospital-acquired	43 (81%)	126 (62%)	
Community-acquired	10 (19%)	76 (38%)	
Bacteremia source			0.005^d^
Catheter-related	16 (30%)	23 (11%)	
Urinary tract	9 (17%)	76 (38%)	
Skin and soft tissue	8 (15%)	16 (8%)	
Respiratory tract	7 (13%)	36 (18%)	
Gastrointestinal tract	5 (9%)	17 (8%)	
Primary	5 (9%)	17 (8%)	
Other	3 (6%)	17 (8%)	
Bacterial isolate			< 0.001^d^
*Klebsiella pneumoniae*	39 (73%)	74 (37%)	
*Enterobacter* spp.	9 (17%)	13 (6%)	
*Serratia marcescens*	2 (4%)	7 (3%)	
*Escherichia coli*	1 (2%)	98 (49%)	
Other bacteria	2 (4%)	10 (5%)	
Critical illness (Pitt bacteremia score ≥ 4)			0.001^c^
Yes	26 (49%)	53 (26%)	
ICU admission			< 0.001^c^
Yes	41 (77%)	100 (50%)	
Underlying diseases and comorbidities			
Surgery			0.008^c^
Yes	26 (49%)	60 (30%)	
Immunosuppression			0.008^c^
Yes	26 (49%)	60 (30%)	
Trauma			0.59^d^
Yes	0 (0%)	5 (2%)	
Renal disease			0.54^c^
Yes	10 (19%)	46 (23%)	
Heart disease			0.08^c^
Yes	12 (23%)	26 (13%)	
Lung disease			0.68^c^
Yes	6 (11%)	19 (9%)	
Liver disease			0.75^d^
Yes	4 (8%)	12 (6%)	
Diabetes			0.17^c^
Yes	7 (13%)	44 (22%)	
Antibiotic treatment			
Empirical treatment (*n* = 231)			< 0.001^c^
At least 1 active antibiotic	11 (22%)	103 (57%)	
No active antibiotic	40 (78%)	77 (43%)	
Change of the empirical regimen after culture report (*n* = 209)			0.43^c^
Yes	35 (74%)	111 (69%)	
No	12 (26%)	51 (31%)	
Definitive treatment (*n* = 228)			< 0.001^d^
At least 1 active antibiotic	37 (77%)	175 (97%)	
No active antibiotic	11(23%)	5 (3%)	
Type of active definitive treatment (*n* = 212)			< 0.001^c^
Combination therapy	29 (78%)	38 (22%)	
Monotherapy	8 (22%)	137 (78%)	
Active definitive treatment regimen (*n* = 212)			0.18^d^
With carbapenem	35 (95%)	150 (86%)	
Without carbapenem	2 (5%)	25 (14%)	

^a^ Data represent No. (%) of patients unless otherwise specified.

^b^ Mann-Whitney *U* test.

^c^ *χ*^*2*^ test.

^d^ Fisher exact test.

The median patient age was 60 years (range 0.1–99) and 145/248 patients (59%) were male. Most of the patients (169/255, 66%) had hospital-acquired infections. The most frequent bacteremia sources were the urinary tract in 85/255 patients (33%), the respiratory tract in 43/255 patients (17%) and catheter-related in 39/255 patients (15%). Most of the community-acquired infections (56/86, 65%) were urinary tract infections. Among all 255 patients, the main microorganisms isolated were *Klebsiella pneumoniae* (113, 44%), *Escherichia coli* (99, 39%), *Enterobacter* spp. (22, 9%), and *Serratia marcescens* (9, 4%). Whereas most of the CPE (39/53, 73%) were *K*. *pneumoniae*, *E*. *coli* was the leading bacteria of non-CPE isolates (98/202, 49%). Overall, 79/255 patients (31%) were critically ill. Critical illness was significantly more frequent in patients with CPE BSI than in those with non-CPE BSI (49% vs. 26%, *p* = 0.001). We found that body temperature, mental status and cardiac arrest were comparable among patient groups; but patients with CPE BSI were more likely to present with hypotension and mechanical ventilation than patients with non-CPE BSI (*p* = 0.001). Regarding underlying diseases, only surgery history and immunosuppression were more likely present in patients with CPE BSI than in patients with non-CPE BSI (49% vs. 30% and 49% vs. 30%, respectively).

### Antibiotic susceptibility testing

*In vitro* non-susceptibility of isolates to all screened antibiotics was higher in the patients with CPE BSI (Table 3). In this group, resistance to ertapenem, imipenem and meropenem was 95% (36/38), 74% (25/34) and 78% (39/50), respectively. The most active drugs were colistin (susceptibility of 96%, 23/24), tigecycline (susceptibility of 79%, 27/34), and amikacin (susceptibility of 78%, 38/49). In the non-CPE BSI group, at least 90% of the isolates were susceptible to amikacin, most carbapenems, colistin and tigecycline.

**Table 3:** Antimicrobial susceptibility in clinical blood isolates among patients with carbapenemase-producing *Enterobacteriaceae* (CPE) bloodstream infection (BSI) and patients with non-CPE BSI.

Antibiotic	Patients with CPE BSI^a^, *n* = 53			Patients with non-CPE BSI^a^, *n* = 202		
	S	I	R	S	I	R
**Aminoglycosides**						
Amikacin	38 (78%)	2 (4%)	9 (18%)	157 (90%)	7 (4%)	10 (6%)
Gentamicin	19 (40%)	0 (0%)	29 (60%)	59 (42%)	4 (3%)	77 (55%)
Ciprofloxacin	10 (18%)	2 (4%)	42 (78%)	55 (29%)	10 (5%)	123 (66%)
**Penicillins and cephalosporins**						
Ampicillin/sulbactam	1 (3%)	0 (0%)	35 (97%)	7 (6%)	5 (5%)	94 (89%)
Ceftriaxone	2 (5%)	0 (0%)	37 (95%)	8 (5%)	7 (4%)	143 (91%)
Cefotaxime	1 (3%)	1 (3%)	33 (94%)	14 (11%)	1 (1%)	116 (88%)
Ceftazidime	1 (2%)	4 (8%)	46 (90%)	14 (8%)	13 (7%)	154 (85%)
Aztreonam	3 (11%)	2 (8%)	21 (81%)	19 (14%)	8 (6%)	112 (80%)
Piperacillin/tazobactam	5 (12%)	2 (5%)	35 (83%)	72 (65%)	5 (5%)	33 (30%)
Cefepime	4 (8%)	3 (6%)	42 (86%)	42 (24%)	15 (9%)	116 (67%)
**Carbapenems**						
Ertapenem	2 (5%)	0 (0%)	36 (95%)	103 (90%)	0 (0%)	11 (10%)
Imipenem	8 (23%)	1 (3%)	25 (74%)	113 (93%)	1 (1%)	7 (6%)
Meropenem	9 (18%)	2 (4%)	39 (78%)	175 (92%)	1 (1%)	14 (7%)
Doripenem	0 (0%)	0 (0%)	9 (100%)	15 (88%)	0 (0%)	2 (12%)
Colistin	23 (96%)	0 (0%)	1 (4%)	43 (98%)	0 (0%)	1 (2%)
Tigecycline	27 (79%)	4 (12%)	3 (9%)	97 (91%)	7 (6%)	3 (3%)

^a^ Data are No. (%) corresponding to antibiotic susceptibility of *Enterobacteriaceae* isolates in both patient groups. S, susceptible; I, intermediate; R, resistant.

### Antibiotic treatment

Out of the 231 patients in whom empirical antibiotic treatment was recorded, 114 (49%) received at least 1 active antibiotic, while 117 (51%) received no active antibiotic. Definitive treatment was given to 228/255 (89%) patients; 212 (93%) of these 228 patients received at least 1 active antibiotic and 16/228 (7%) received a non-active antibiotic therapy. Comparison of treatment variables among patients with CPE BSI and patients with non-CPE BSI is shown in Table 2. The proportion of empirical and definitive active antibiotic treatment was significantly higher in the patients with non-CPE BSI (*p* < 0.001). Combination therapy was significantly more frequent in the CPE BSI group than in the non-CPE BSI group (*p* < 0.001). There were no statistically significant differences in the change of the empirical antibiotic regimen after culture report and the use of a carbapenem in the definitive regimen among study groups. The patients with CPE BSI who received definitive antibiotic treatment were 48/53 (91%) (Table 4) but only 37 of these 48 received an active antibiotic regimen. While 29/37 (78%) received a combination therapy, 8/37 (22%) received monotherapy. A carbapenem was part of the regimen in 35 (95%) of those 37 patients. Among the patients who received active combination therapy, the most common regimen was carbapenem + colistin or polymyxin B (11/29, 38%).

**Table 4:** Outcome of patients with carbapenemase-producing *Enterobacteriaceae* bloodstream infection according to treatment regimen.

Definitive antibiotic treatment	Total of patients	Mortality, No. (%)
Active	37	22/37 (60%)
Combination therapy	29	17/29 (59%)
Carbapenem-containing regimen	28	
Carbapenem + colistin or polymyxin B	11	
Carbapenem + colistin or polymyxin B + tigecycline	7	
Carbapenem + colistin or polymyxin B + aminoglycoside	4	
Carbapenem + tigecycline	3	
Carbapenem + aminoglycoside + polymyxin B + tigecycline	1	
Carbapenem + aminoglycoside + tigecycline	1	
Carbapenem + rifampin	1	
Carbapenem-sparing regimen	1	
Polymyxin B + rifampin	1	
Monotherapy	8	5/8 (63%)
Carbapenem	7	
Tigecycline	1	
No active	11	8/11 (73%)

### Mortality and survival

The overall mortality was 37% (94/255). Mortality was significantly higher in patients with CPE BSI than in those with non-CPE BSI (64% vs. 30%, *p* < 0.001). Mortality was attributable to BSI in 55/94 dead individuals (59%). Attributable mortality was significantly higher in the patients with CPE BSI (85% vs. 43%, *p* < 0.001). In the CPE BSI group, the mortality of patients who received an active antibiotic regimen did not statistically differ from the mortality of the patients without an active antibiotic therapy (22/37 vs. 8/11, *p* = 0.5). Mortality in monotherapy group was similar to mortality in the combination therapy group (5/8 vs. 17/29, *p* = 1.0).

**Fig 1:**
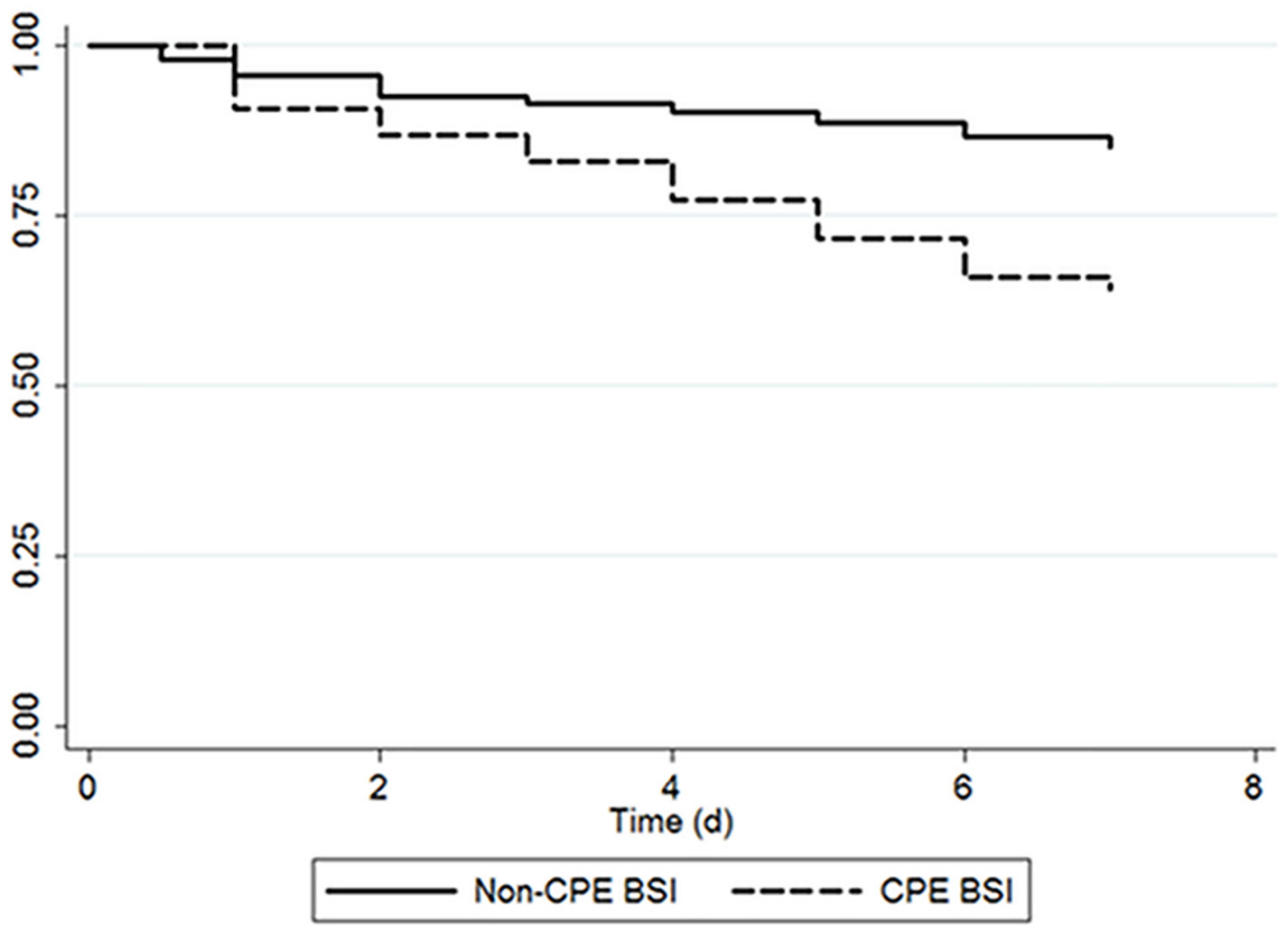
Kaplan-Meier survival estimates at 7 days of patients with carbapenemase-producing *Enterobacteriaceae* (CPE) bloodstream infection (BSI) (dashed line) vs. non-CPE BSI (solid line). *p* < 0.001 (log rank test).

No significant difference was found between subjects with CPE BSI and subjects with non-CPE BSI regarding Kaplan-Meier survival analysis at 72 hours (*p =* 0.07). In contrast, there was a significantly greater survival in non-critical patients than in critical patients at 72 hours (*p* < 0.0001). Kaplan-Meier survival estimates at 7 days were significantly better for patients with non-CPE BSI than for those with CPE BSI (*p* < 0.001) (Fig 1). Also, there was a statistically significant difference in survival of the patients at day 7 according to critical or non-critical illness (*p* < 0.0001). Similarly, significant survival differences were found at 28 days for these outcomes (*p* < 0.0001).

In multivariate analysis (Table 5), we found that CPE BSI (adjusted OR [aOR] 4; 95% CI 1.7–9.5; *p* = 0.002) and critical illness (aOR 6.5; 95% CI 3.1–13.7; *p* < 0.001) were independently associated with in-hospital mortality.

**Table 5:** Risk factors associated with mortality among patients with carbapenemase-producing *Enterobacteriaceae* bloodstream infection.

	Multivariate analysis	
Variable	aOR (95% CI)	*p* value
Critical illness (Pitt bacteremia score ≥ 4)	6.5 (3.1–13.7)	< 0.001
Change of the empirical regimen after culture report	1.8 (0.8–4.1)	0.15
Definitive treatment with carbapenem	1.2 (0.4–3.7)	0.77
Carbapenemase-producing *Enterobacteriaceae* bloodstream infection	4 (1.7–9.5)	0.002

aOR, adjusted odds ratio.

## Discussion

Since 1990s, CRE have been reported worldwide [1,32,33]. CRE poses a public health threat given their alarming endemicity in many world regions, including Latin America [5], and its association with worse clinical and economic outcomes [1,2,8]. In this Latin American study of patients with BSI caused by *Enterobacteriaceae*, we described the clinical characteristics and determined the risk factors associated with in-hospital mortality. Of the 255 *Enterobacteriaceae* isolates included, 53 were found by PCR to harbor KPC, VIM or NDM. The molecular characterization performed to the isolates was essential because the phenotype reported by automated systems routinely used in microbiology laboratories might not detect up to 6–87% of the β-lactamases associated with carbapenem resistance in known resistant isolates [34]. As many CPE just have discretely elevated MICs [35], PCR was a reliable technique for the classification of the patient groups in this study.

KPC is the most prevalent Ambler class A carbapenemase worldwide and has the biggest clinical significance [5,10]. In this study, most CPE (44/53, 83%) were KPC producers, and the majority of the CPE isolates (39/53, 73%) corresponded to *K*. *pneumoniae*, which is the most frequently identified carbapenemase producer. This finding is consistent with other studies [1,5].

As expected, CPE isolates presented a higher *in vitro* resistant profile to all antibiotics. Among the carbapenems, resistance to ertapenem was present in 95% of the strains, while resistance rate to imipenem and meropenem was 74% and 78%, respectively. CPE isolates tested for doripenem exhibited 100% resistance although the interpretation was limited given the small number of isolates tested (*n* = 9). Colistin, tigecycline and amikacin had the best *in vitro* activity against CPE, with a susceptibility proportion of 96%, 79% and 78%, respectively.

Regarding the comparison of the two patient groups, we found significant differences on variables such as critical illness, ICU admission, surgery history and immunosuppression, which were more likely present in patients with CPE BSI. Prior studies have identified associations between infection by CPE, predominantly *K*. *pneumoniae*, and in-hospital factors, such as ICU stay or admission [6,36], surgery [36], length of stay [2], mechanical ventilation [2,36], central venous catheters [6], history of exposure to antipseudomonal penicillins, cephalosporins, fluoroquinolones or carbapenems [2,6,13,25,36,37], recent solid organ or stem-cell transplant [2], and higher illness severity [6,25].

Both the proportions of empirical and definitive active antibiotic treatment were significantly lower in patients with CPE BSI, likely due to the few antibiotic options for which CPE are susceptible. For the same reason, combination therapy was likely found to be more frequent in patients with CPE BSI. Among 48 patients who received definitive antibiotic treatment, 29 received an active combination therapy. Carbapenems were used in 28 (97%) of these antibiotic regimens.

Mortality in patients with CPE BSI was 64% which is similar to other studies carried out in the USA and Europe [8,11–17,20,21]. Only one study addressing CPE infections has been performed in Latin America, in which 118 patients infected with KPC-producing *Enterobacteriaceae* were included; 78 patients of these presented bacteremia and the 30-day mortality was 50% [18]. In our study, in which all patients had bacteremia not exclusively caused by CPE, we found that both overall and attributable mortality rates were higher in patients with CPE (*p* < 0.001).

Patients who received monotherapy and patients who received combination therapy had similar mortality rates (63% vs. 59%; *p* = 1.0). This finding is interesting as other research works on patients infected with CRE elsewhere have demonstrated lower mortality when treated with combination therapy instead of monotherapy [15,16,38]. In our study, we probably found a non-statistically significant association due to the low number of patients with CPE BSI (*n* = 53) and also due to the study design. However, this is a subject of wide debate and stands yet as an unresolved question.

In our study, the survival analyses clearly showed that the adverse outcome likely occurred more frequently at 7 days after bacteremia onset among patients with CPE BSI and among critically ill patients. Although Kaplan-Meier estimates for survival were also significantly higher in patients with non-CPE BSI even at 28 days, analysis at that time is not the most appropriate because several causes not evaluated in our study could lead to in-hospital death.

Although we were mainly interested in determining if CPE BSI was a risk factor for mortality, we first assessed the presence of underlying diseases and comorbidities in the patients with CPE BSI and patients with non-CPE BSI. Most underlying diseases were comparable between both groups; however, critical illness (PBS ≥ 4) was statistically significant higher in the CPE BSI group. We found that patients presented significantly more hypotension and mechanical ventilation although the other PBS parameters were comparable among patient groups. After multivariate adjustment, CPE BSI and critical illness were statistically significant factors associated with in-hospital mortality. This indicates that among patients with BSI, those caused by CPE and those with critical illness are independently at a higher risk of death during hospitalization.

There are some limitations in this study to discuss. First, 431/686 (63%) of the isolates received were excluded because they did not meet the selection criteria; this could cause some undetermined bias in our results. Second, different hospitals had no susceptibility testing of the isolates for colistin and polymyxin B, limiting our conclusions on their effectiveness, considering that these antibiotics are often the last therapeutic option for CRE infections. Third, despite that empirical and definitive treatments were assessed in our study, time to the antibiotic infusion and removal of the infection source (i.e., catheter removal, drainage, and debridement) were not evaluated. Patel *et al* showed higher survival in patients infected with carbapenem-resistant *K*. *pneumoniae* treated with antibiotics and removal of the focus, compared with those treated with antibiotics alone, although they did not adjust for illness severity [2].

In conclusion, this study provides valuable regional data on the characteristics and clinical impact on mortality of patients with CPE BSI from high-complexity hospitals of Latin America. We confirm that CPE infection is an independent mortality predictor which could encourage healthcare settings to carry out campaigns on prevention, detection, control and treatment of CPE BSI. Finally, we highlight the need to improve actions towards antimicrobial stewardship practices, and increase awareness on CPE among all the public health decision-makers.
